# Evaluating the Quality of Systematic Reviews on Pediatric Sedation in Dentistry: An Umbrella Review

**DOI:** 10.3390/jcm13123544

**Published:** 2024-06-17

**Authors:** Carolina Marques, Mafalda Dinis, Vanessa Machado, João Botelho, Luísa Bandeira Lopes

**Affiliations:** Egas Moniz Center for Interdisciplinary Research Center (CiiEM), Egas Moniz School of Health and Science, 2829-511 Almada, Portugal; carolinascmarques11@gmail.com (C.M.); mafaldinhadinis@gmail.com (M.D.); vmachado@egasmoniz.edu.pt (V.M.); luisabpmlopes@gmail.com (L.B.L.)

**Keywords:** conscious sedation, sedation, pediatric, paediatric, systematic review, meta-analysis, pediatric dental procedure, procedural sedation, umbrella review

## Abstract

Sedation is a depression of a patient’s state of consciousness, induced by medications, that can reach different levels of intensity during a medical procedure. Conscious sedation produces a minimally depressed level of consciousness without impairment of the ability to maintain an open airway, of protective reflexes or of responses to verbal and physical stimulation. This umbrella review is aimed at critically assessing the available systematic reviews (SRs) and meta-analyses (MA) on sedation in children/adolescents. An electronic database search was conducted that included Pubmed-Medline, Web of Science, Cochrane, Scopus, Scielo, Embase, LILACS and TRIP and the scope of which extended until January 2023. The risk of bias (RoB) of SRs was analyzed using the Measurement Tool to Assess SRs criteria 2 (AMSTAR2). Of 998 entries, 37 SRs were included. In terms of methodological quality, eight studies were assessed as having critically low quality, four studies had low quality, nine studies had moderate quality, and sixteen were considered to be of high quality. Based on the current guidelines, the most employed drugs in pediatric dentistry for sedation are nitrous oxide and midazolam; however, the available evidence supporting their use is insufficient and of low/critically low quality. The combined technique is recommended (nitrous oxide (30–50%) + midazolam). The optimal dose of oral midazolam is 0.75 mg/kg. The level of methodological quality of SRs is expected to increase according to the results and future directions of this umbrella review.

## 1. Introduction

Pediatric dentists may face difficulties managing fear and anxiety in children [1]. These ultimately affect the success, quality and safety of dental treatment [2]. The global prevalence of dental anxiety in children ranges from 3% to 43% [3], with emotional factors involved that may jeopardize its management, namely pain, fear of the unknown and anger [4].

When treating pediatric patients suffering from dental anxiety, dentists are inevitably exposed to increased stress [5], with more time-consuming treatments, increased costs and other difficulties encountered during dental practice [6]. To this end, sedation is an appropriate and acceptable alternative to general anesthesia in order to reduce the child’s anxiety. As an alternative to general anesthesia, sedation requires appropriate resources and location [2,7].

Additionally, as there is no consensus regarding conscious sedation techniques or the most appropriate sedation approach depending on dental fear/anxiety or non-cooperative/medically compromised children during dental treatment, we highlight the importance of this study.

Sedation is a combination of sedative and anesthetic drugs administrated during a medical procedure. Patients undergo a minimally depressed consciousness without ability impairment, maintaining an open airway, protective reflexes and responses to verbal and physical stimulation [8]. Sedation is highly effective in mitigating anxiety and pain perception, allows for improved cooperation and contributes to high quality and safe care [8]. The choice of sedation technique depends on the patient’s needs and health status, the type of surgical procedure to be performed, and the surgeon’s preference [9]. The ideal sedative agent for use in the outpatient setting should provide a rapid onset of action and stable operating conditions, be easily reversed, ensure rapid and a predictable recovery, and have limited side effects [9]. 

Currently, commonly used sedative agents are administered together or alone, through different routes and dosages in pediatric dental settings [2], which can make it difficult for the dentist to decide the best technique and the ideal sedative [10]. 

Public health bodies have been providing guidance on the recommended sedation techniques and drugs, guiding practitioners in making informed clinical decisions with better outcomes. The follow-up of such guidance is often supported by evidence-based studies, which require continuous revision to ensure accuracy and temporal relevancy. This umbrella review assesses all systematic reviews (SRs) ever produced on sedation in children/adolescents with a specific emphasis on a drug’s success rate and the quality of evidence.

## 2. Materials and Methods

We report this umbrella review using the Preferred Reporting Items for Systematic Reviews and Meta-Analyses (PRISMA) guideline updated in 2020. The review protocol was approved a priori by all authors.

The review question was as follows: “What is the level of evidence for the efficacy and safety of sedation for children with fear and/or anxiety and uncooperative/medically compromised children undergoing dental treatment?”

The following PECO statements were set: Population (P)—Children with fear and/or children anxious about undergoing dental treatment; Exposure (E)—Sedation; Comparison (C)—Placebo or without sedation; and Outcome (O)—Behavioral, anxiolytic efficacy and safety.

### 2.1. Eligibility Criteria

The inclusion criteria were as follows: (1) is a systematic review or a meta-analysis; (2) retrieves its data from human studies; and (3) investigates the performance of sedation undergoing clinical procedures. No restrictions to publication year or language were applied. Grey literature was searched through two appropriate databases and registers (https://www.ntis.gov/, www.apa.org/pubs/databases/psycextra, accessed on 21 May 2024).

### 2.2. Information Sources Search

An electronic data search was performed in eight electronic databases: PubMed (via Medline), Web of Science, Scopus, Cochrane Database of Systematic Reviews, Scientific Electronic Library Online (Scielo), The Excerpta Medica Database (EMBASE), Latin-American scientific literature in health sciences (LILACS), and Turning Research Into Practice (TRIP). We merged keywords and subject headings in accordance with the thesaurus of each database and applied exploded subject headings, with the following syntax (“conscious sedation” [MeSH] OR sedation OR “conscious sedation”) AND (pediatric [MeSH] OR paediatric OR pediatric) AND (systematic review).

### 2.3. Study Selection

Two researchers (CM and MD) independently screened titles and abstracts. The agreement between the reviewers was assessed by Kappa statistics. Any paper classified as potentially eligible by either reviewer was ordered as a full-text and independently screened by the reviewers. All disagreements were resolved through discussion with a third reviewer (LBL).

### 2.4. Data Extraction Process and Data Items

Two researchers (CM and MD) independently extracted the following: authors and year of publication, objective/focused question, databases searched, number of studies included, type of studies included, main results and main conclusions. All disagreements were resolved through discussion with a third reviewer (LBL).

### 2.5. Risk of Bias Assessment

Two researchers (CM and LBL) employed the Measurement Tool to Assess Systematic Reviews (AMSTAR 2) to determine the methodological quality of the included reviews.

AMSTAR 2 is a comprehensive, 16-item tool that rates the overall confidence of the results of the review. We chose AMSTAR2 due to its comprehensive evaluation of methodological quality, validation and wide acceptance. According to the AMSTAR guidelines, the quality of the systematic reviews was considered as follows: high means ’zero or one non-critical weakness;´ moderate means ´more than one non-critical weakness;´ low means ´one critical flaw with or without non-critical weaknesses;´ and critically low means ´more than one critical flaw with or without non-critical weaknesses.’ The estimation of the AMSTAR quality rate for each study was calculated through the AMSTAR 2 online tool (https://amstar.ca/Amstar_Checklist.php, accessed on 21 May 2024).

## 3. Results

### 3.1. Study Selection

**Figure 1:**
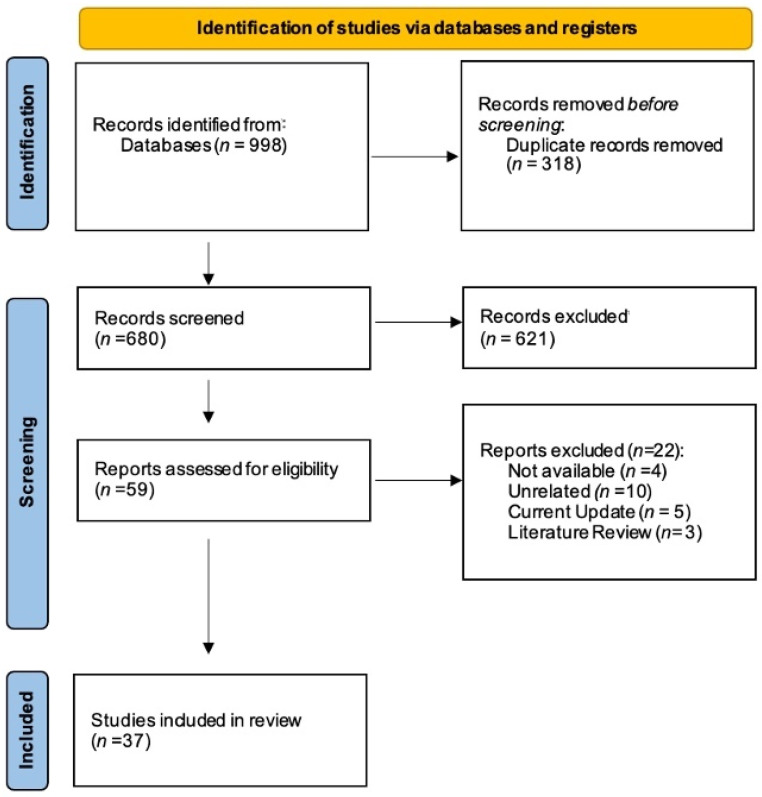
PRISMA flowchart of studies.

Electronic searches retrieved a total of 998 titles through the database search. After manual assessment of title/abstract and removal of 318 duplicates, 59 potentially eligible full texts were screened. Full-text screening excluded 22 studies with reasons, resulting in 37 systematic reviews that fulfilled the inclusion criteria (Figure 1). The level of agreement was considered excellent (k = 1.00). Further information regarding reasons for SR exclusion is available in the Supplementary Materials.

### 3.2. Studies’ Characteristics

Overall, 25 SRs with meta-analysis and 12 SRs without meta-analysis were included (Table 1). Multiple sub-topics were investigated; for example, a comparison of the sedation efficacy of different sedative drugs, dosages and administration routes.

The methodological characteristics are detailed in Table 1. All SRs covered a defined timeframe; however, five did not mention such information. Four SRs failed to report a language restriction, fourteen restricted their search to studies in English, four restricted their search to studies in English and Chinese, one restricted their search to studies in English, Spanish and Italian, and fourteen had no language restriction.

### 3.3. Methodological Quality

Regarding the methodological quality of SRs, eight studies were assessed as of critically low quality (22%), four as of low quality (11%), nine studies as of moderate quality (24%), and sixteen as of high quality (43%) (detailed in Table 2). In conclusion, 67% of the SRs were assessed as having moderate/high quality studies. None of the included SR fully complied with the AMSTAR2 checklist. Overall, SRs mostly failed in terms of the following: reporting on the sources of funding for the studies included in the review (100%, *n* = 37); satisfactory technique for assessing the risk of bias (27%, *n* = 10); providing a satisfactory explanation for, and discussion of, any heterogeneity observed in the results (21.6%, *n* = 8); studying selection performed in duplicate (18.9%; *n* = 7); and review methods established a priori (18.9%, *n* = 7).

### 3.4. Synthesis of Results

#### 3.4.1. Active Principle

##### Nitrous Oxide

Critically low: N_2_O is an effective sedative/analgesic for mildly to moderately painful pediatric procedures and does not have major side effects (5% nausea and vomiting) [44].

Low: The estimated efficacy rates of NOIS (≤50% N_2_O) were 94.9% globally and 91.9% in children [20].

High: Comparing the combination technique NOIS (30–50% N_2_O) plus midazolam or midazolam alone (0.05–0.6 mg), a statistically significant improvement in the overall cooperation between the interventions tested was not found, with high quality [39]. Nonetheless, the use of the combination technique rather than the individual use is recommended because of the reduction in recovery time, the dose of midazolam and the subsequently lower risk of adverse effects [39].

##### Midazolam

Critically low: Midazolam is a safe and effective drug [12,29], with an adequate safety profile for use in minimal sedation [29]. Additionally, when compared with a placebo, it provides adequate sedation faster, and improves mental attitude and hypnotic and motor activity, and the quality of sedation and dental treatment [37]. The combination with hydroxyzine results in safe and effective sedation, and might be more advantageous, resulting in less crying and movement [29].

High: Oral midazolam (0.25–1 mg/kg) is effective and safe for pediatric sedation [2,21] and is associated with more cooperative behavior when compared with a placebo [2]. Furthermore, the combination with Meperidine appears to enhance the effectiveness and duration of action when managing pediatric patients [21].

Midazolam vs. Ketamine

Critically low: Oral midazolam may be preferable because of the shorter recovery and lower cost [29].

Moderate: Intranasal ketamine has been reported to offer a rapid onset of sedation, and the highest overall success (89%) when compared with intranasal midazolam and the combination of the two drugs [33].

High: Both are considered safe and effective in moderate sedation [14]. Intranasal midazolam provides more rapid onset and recovery; however, it has only sedative properties. Meanwhile, ketamine provides better sedation, anxiolysis and analgesia when used for premedication [14].

Midazolam vs. Chloral hydrate

High: Oral midazolam has an increased level of sedation [21].

Midazolam/Ketamine vs. Midazolam/Sufentanil

Moderate: Both were equally effective in terms of sedation [33].

Midazolam vs. Melatonin

Moderate: Premedication with oral midazolam in pediatric patients is superior, with a higher satisfaction among parents and operators [16].

##### Dexmedetomidine

Critically low: Dexmedetomidine is safe and effective when used for sedation in pediatric patients undergoing dental procedures [40].

Moderate: Dexmedetomidine is safe and effective agent for procedural sedation, and has shown to induce a reduction in pulse rate and systolic blood pressure, greater intra/post-operative analgesia, and better overall success rate; however, it has a longer onset of sedation and recovery time when compared with midazolam and ketamine [24,45,46].

Dexmedetomidine vs. Ketamine

High: Both provide comparable sedation for pediatric dental surgery [30]. Additionally, there are no significant impacts on intraoperative and postoperative analgesia scores, blood pressure and oxygen saturation; however, dexmedetomidine is associated with decreased heart rate, and systolic blood pressure, which also results in a longer recovery time [30].

Dexmedetomidine vs. Midazolam plus Propofol

Critically low: Midazolam plus propofol is considered less safe and effective [28]. Also, dexmedetomidine premedication provides shorter recovery time, less incidence of side effects, more analgesic effects postoperatively, higher mean arterial pressure, and longer onset of sedation [40].

Dexmedetomidine vs. Midazolam

Low: Both are equally effective in pediatric dentistry [18]. There are no significant differences in behavior, parental separation, or mask acceptance. On the other hand, dexmedetomidine shows a wider margin of safety and a lower incidence of emergence delirium [18]. The success rate was higher in children treated with dexmedetomidine [25].

Moderate: Dexmedetomidine provides more satisfactory sedation upon parent separation and mask acceptance, reduces agitation, delirium and shivering during the postoperative period, reduces the request for rescue analgesia and has a prolonged onset of sedation [42,47]. On the other hand, there are no significant differences in parental separation, anxiety, mask acceptance or the emergence of delirium between both [42,48].

High: Dexmedetomidine provides effective sedation [36], associated with more satisfactory separation from parents [11,18,23,34,35,41,43,47] and mask acceptance [11,34,35,41,43,47]. It leads to a lower incidence of agitation, postoperative delirium, and tremors, reduces the need for analgesia [11,23,34,35,41,43,47], and has a prolonged onset of sedation [11,34,35,41,43,47]. It also decreases heart rate, systolic blood pressure [11], and nasal irritation [35]. However, the studies also report that there are no significant differences at the time of separation from parents [34,41,43,48], mask acceptance [18,23,34,41,43,48], incidence of agitation, postoperative delirium, tremors, need for analgesia [34,41,43,48], onset of action, and sedation [18]. Nevertheless, one SR cannot be used to draw an overall conclusion on this topic because of limited RCTs [4].

##### Ketamine

Critically low: The use of ketamine (alone or in combination) can provide safe, effective and timely sedation in pediatric patients regardless of the route of administration [32].

##### Melatonin

Moderate: It is similar to placebo and was not shown to contribute to the N_2_O/O_2_ sedation of anxious children [16].

##### Oral Chloral Hydrate

Moderate: The sedation success rate during dental examinations was 76.05% [26]. When compared with a placebo, the success rate of sedation increased significantly [26]. There was no significant difference in success rate of sedation, when compared with a diazepam and barbiturates group [26].

##### Propofol

Critically low: Propofol could be considered for sedation in pediatric procedures, and may be an excellent alternative with the shortest recovery, the absence of nausea and vomiting, reasonable surgical satisfaction and is highly effective in terms of onset of sedation [28].

High: Propofol could be an excellent alternative, with the shortest recovery, absence of nausea and vomiting and reasonable surgical satisfaction [19].

##### Ketamine/Propofol (Ketofol)

Ketofol vs. Propofol

Low: Ketofol provides effective sedation levels without serious perioperative complications [22]. It is associated with lower complications, like stable cardiovascular hemodynamics and shorter recovery time, and higher satisfaction rates in pediatric patients undergoing dental treatment [22].

High: Both exhibited a similar sedation profile, while propofol emerged as a safer option due to the lesser incidence of respiratory depression and desaturation [19].

Ketofol vs. Ketamine

Moderate: Ketofol produces stable cardiovascular hemodynamics, lower psychomimetic and respiratory adverse events, has a very good security, higher satisfaction and shorter recovery time, as well as a lower incidence of nausea and vomiting [27].

Ketofol vs. Ketamine/Dexmedetomidine

High/Critically low: Ketofol might be a better option, due to lower vomiting and nausea episodes and higher satisfaction levels [19,28].

#### 3.4.2. Dosages

SRs reported information for midazolam and Dexmedetomidine for oral and intranasal routes, respectively (Table 3).

#### 3.4.3. Administration Route

##### Oral

Critically low: This route is advocated for short dental procedures; however, it carries a risk of over sedation, and has a longer recovery time and a longer duration of onset [12].

Moderate: There is no statistically significant difference between oral and intranasal routes on behavior and sedation level [13].

##### Intranasal

Midazolam

Critically low: It has a duration onset three times faster and a rapid absorption, when compared with oral route [12].

Moderate: There is no statistically significant difference between intranasal and other routes on the outcome of behavior and sedation level [1].

High: Children showed signs of nasal irritation [11,23,36,47].

Ketamine

Moderate: The administration is well tolerated and without serious adverse effects [38]. Additionally, when compared with other drugs and routes, the effectiveness of sedation with respect to superiority was inconsistent [38].

Dexmedetomidine

Moderate: It allows for a better onset of sedation, an adequate depth of sedation, a better ease of treatment completion, a longer recovery time and a better treatment outcome, when compared with the oral route [24,45].

High: It can provide equally efficacious and satisfactory sedation with fewer events and fast recovery. Moreover, a nebulized combination of low dose ketamine produced more satisfactory sedation and better postoperative analgesia, with a more rapid recovery and with no significant adverse effects. When compared with oral midazolam, dexmedetomidine provides a higher success rate in sedation and parental separation [15].

##### Sublingual

Midazolam

Critically low: This route contributes to high absorption [12].

##### Intravenous

Midazolam

Critically low: This route permit a gradual adjustment of midazolam and allows one to control the level of sedation [12].

##### Rectal

Critically low: Appears to be feasible, moderately effective and safe. The adverse events reported were amnesia/drowsiness, desaturation, aggressiveness, tiredness and agitation [49].

#### 3.4.4. Adverse Events

Most SRs reported adverse effects [2,4,11,15,16,18,19,21,22,23,27,28,29,33,34,35,36,38,39,41,42,43,44,49], while twelve did not [1,12,13,17,20,24,25,26,30,32,37,40]. We have summarized the most common and the least common adverse effects (Table 4).

## 4. Discussion

### 4.1. Summary of the Main Results

The present systematic review evaluated a total of 37 systematic reviews on sedation in children/adolescents, focusing on the success rate of the drug and the quality of the evidence. In order to provide readers with a deeper and more reliable understanding of recent advances in this scientific field, we have chosen to extrapolate the high-level evidence findings, in order to relate them to current clinical practice and recommendations. Thus, 16 SRs that are supported by conclusions with high quality evidence are highlighted. Five of these were primarily focused on dentistry. In summary, the most employed drugs in pediatric dentistry for sedation are nitrous oxide and midazolam, and the combined technique is recommended (nitrous oxide (30–50%) + midazolam). The optimal dose of oral midazolam is 0.75 mg/kg. It should be noted that procedural sedation and analgesia are currently applied. This involves the administration of sedative or dissociative agents so as to reduce discomfort and anxiety and to manage pain and potentially unpleasant memories during diagnostic and therapeutic dental procedures. This facilitates painful procedures while ensuring safety and comfort, without compromising the airways [22,50,51,52,53].

In contrast, conscious sedation is used to control behavior during pediatric dental rehabilitation, reduce anxiety, improve patient behavior, and maximize the potential for amnesia, in which the patient experiences a state of depressed consciousness while remaining responsive to verbal requests or tactile stimuli [12,51].

Regarding midazolam, the results are inconsistent and are supported by different levels of evidence, rendering them insufficient. The optimal dose of oral midazolam, in terms of efficacy, acceptability, and safety in pediatric dentistry, seems to be 0.75 mg/kg [21]. Additionally, its combination with meperidine seems to increase efficacy and duration of action [21].

Midazolam was also compared with other sedative drugs, such as ketamine, dexmedetomidine, and chloral hydrate. This was undertaken in order to investigate safety and sedation efficacy. It has been reported to have an increased level of sedation when compared with chloral hydrate [21] and is equally safe and effective when compared with ketamine [14]. Additionally, when compared with dexmedetomidine, the results are inconsistent; there is not exactly a consensus.

On the other hand, when comparing dexmedetomidine with ketamine, both provide identical sedation in pediatric dentistry. However, dexmedetomidine is associated with a decrease in heart rate, systolic blood pressure, and results in a longer recovery time [30].

Its intranasal administration, when compared with the oral route, midazolam provides a higher success rate in sedation and, at the time of parental separation, offers fewer adverse effects and faster recovery [15]. In addition, a low-dose combination with ketamine produces more satisfactory sedation and better postoperative analgesia, with faster recovery and no significant adverse effects [15].

Regarding propofol alone, or its comparison with ketofol, the results offer high to very low evidence, proving to be inconsistent and insufficient. The same occurs for the comparison of ketofol with ketamine–dexmedetomidine.

Although studies compare adverse effects that are generally very minimal, it is notable that the most common general adverse effects were nausea [14,19,21,23,34,35,41,43], vomiting [2,4,15,19,23,35], tremor [23,34,35,41,43], and desaturation [15,19,23]. Other events were reported less frequently, namely drowsiness, respiratory events, diplopia [2,21], hypoxia, coughing [2,19], dysphoria [21], apnea, stridor, laryngospasm [19], hallucinations, amnesia, hiccups, enuresis, bronchospasm, hypersalivation, otalgia, epistaxis, hyperexcitation, uncontrollable behavior, malaise, irritability, crying, and sneezing [2]. Adverse effects, such as nasal irritation [4,11,23,34,35,41,43] and teary eyes [4,11,23,34,35,41,43], have been associated with the administration of intranasal midazolam.

### 4.2. Evidence Quality and Potential Bias in the Review Process

Around 67% of the studies showed moderate/high quality evidence and 43% were of a high quality. Focusing mainly on the field of dentistry, we found that of the 11 SRs included, only 46% were of high methodological quality, rising to 64% when considering moderate/high evidence, according to AMSTAR-2.

Statistically, the included SRs had, on average, relatively few included studies (mean = 6) and few participants (mean = 508).

A total of 70% of the SRs focused on pediatric sedation in general, and not only in pediatric dentistry, limiting the number of studies that could be retrieved for analysis, resulting in some degree of bias. Future investigations in pediatric dentistry are needed, with a larger sample size and a larger number of studies included; therefore, more randomized controlled trials that compare the effects of drug sedations and their dosages would be helpful for clinical practice. In addition, efforts should be made to establish more conclusive inferences.

### 4.3. Agreements and Disagreements with Other Reviews or Studies

Currently, there are several guidelines and recommendations worldwide that provide guidance for the practice of sedation in pediatric dentistry. However, these recommendations may vary depending on the country, region, or organization. They generally address aspects such as recommended drugs, routes of administration, and dosages, and they are subject to periodic review as new scientific evidence emerges and clinical practices evolve.

Detailed information was collected from 11 previously selected guidelines, covering several countries such as, Chile [54], Singapore [55], Scotland [56], China [57], Canada [58], Australia [59], Japan [52], the United Kingdom [50], and the United States [51]. There is one that covers the European continent [8].

All, except the Japanese guideline [52], consider and recommend the administration of nitrous oxide; however, according to the results obtained, it was found that the efficacy rate of the drug is supported by low to very low evidence, converging with what the European [8] guideline states.

Although there is some divergence on this topic, the American [51] and Canadian [58] guidelines converge in recommending the use of the combined technique (nitrous oxide + midazolam) and are supported by high quality evidence, according to the results.

In addition, the American [51] guideline mentions that low doses of chloral hydrate (10–25 mg/kg), in combination with other sedative medications, are commonly used. The South African [53] guideline agrees with the previous recommendation, but does not recommend the combination with other sedatives, indicating the prescription of a higher dose (25–100 mg/kg).

However, we found that the 76.05% success rate of the drug is only supported by moderate evidence, and that oral midazolam is preferable, with an increased level of sedation, according to high evidence results. These aspects are in line with the European [8], Canadian [58] and United Kingdom [50] guidelines, which do not recommend the use of chloral hydrate.

The South African [53], European [8], Scottish [56], Canadian [58] and UK [50] guidelines recommend the administration of midazolam; however this is supported by a high/very low quality of evidence, making the results inconsistent, and the evidence insufficient.

The recommended dosages differ between guidelines, as well as the routes of administration, and there is no consensus. However, the recommended dosage of 0.3 to 0.5 mg/kg is considered to have very low evidence in providing adequate sedation. Additionally, the 0.75 mg/kg dosage is supported by a high quality of evidence and is considered to be optimal in terms of efficacy, acceptability, and safety in pediatric dentistry.

Considering the routes, these are supported by low/very low-quality evidence, with moderate evidence reporting that there are no statistically significant differences between the oral and intranasal route with regard to behavior and level of sedation. It is important to mention that the routes of administration are described in different guidelines and are not addressed in the American, Japanese, Singapore or Australian guidelines. It was additionally found that the routes of administration of several drugs involved intranasal, buccal, and oral routes, which differ from the approved routes favored by most regulatory bodies in many countries (intravenous or intramuscular). Based on our results, the level of evidence for the intravenous route is inconsistent, being supported by varying levels of evidence, while the intramuscular route is supported by a very low level of evidence.

In the present scenario, an intriguing question arises regarding the routes of administration highlighted in medical guidelines, as many of these lack a solid foundation of high scientific evidence. This gap should not be interpreted as a complete denial of the benefits but rather as an incentive to conduct more studies that support and strengthen their use. Therefore, it is essential to invest in additional, well designed, and rigorous clinical trials to support the use of these routes of administration for sedation.

There are guidelines that recommend the use of another benzodiazepine, diazepam, such as the Canadian [58], Japanese [52] and UK [50] guidelines. However, according to the umbrella review, there were no major results for diazepam in the included SRs, only moderate evidence that there is no significant difference when compared with chloral hydrate.

The European [8] guideline also states that there is insufficient evidence regarding the efficacy of ketamine. This topic meets our results, since they are inconsistent. Finally, the administration of meperidine is not advised; however, this diverges from the results, since the combination of meperidine with midazolam is supported with high evidence, increasing the efficacy and duration of action.

The guidelines do not mention drugs such as propofol, dexmedetomidine, and ketofol, which are not commonly used in the practice of sedation in pediatric dentistry. The results are also inconsistent, and there is not exactly a consensus.

Regarding the adverse effects most often reported by the guidelines, these were respiratory depression, vomiting, airway obstruction, loss of consciousness, airway compromise, desaturation, apnea, nausea, allergic reactions, and hallucinations. In comparison with our results, we find that three of the most frequent adverse events match the guidelines, such as vomiting, nausea, and desaturation. Others are also reported, but at a lower frequency.

In summary, the most reported sedative drugs according to the guidelines are nitrous oxide and midazolam, by different routes and dosages. However, these recommendations are inconsistent with the results presented, since nitrous oxide, although recommended, has a low to very low quality of evidence, and midazolam is reported with high/very low quality of evidence, making it inconsistent and therefore unfit to draw any conclusions from.

Currently, guidelines play a crucial role in guiding healthcare professionals, directly influencing treatment decisions.

According to the current scenario, an intriguing question arises about the drugs highlighted in medical guidelines, as many of them lack a solid base of high scientific evidence. Such gaps raise questions about the validity and reliability of these recommendations, suggesting the possibility that such guidelines, are increasingly based on clinical practice.

The lack of robust evidence can be attributed to factors such as small number of high-quality clinical trials, the lack of adequate funding, or the complexity of clinical trials needed to evaluate the efficacy and side effects of drugs.

However, this gap should not be interpreted as a complete denial of their benefits, but rather as a stimulus for further studies to support and strengthen their use. Thus, it is critical to invest in additional, well designed, rigorous clinical trials that fill this gap in knowledge, and provide robust evidence to support the use of these drugs in sedation.

It is also important that there be a cautious and individualized approach to prescribing these drugs, and that there should be a careful assessment of the risks and benefits, considering clinical experience and the information already available; therefore, the importance of this topic from the point of view of the clinician in a pediatric dentistry environment is significant.

## 5. Conclusions

Sedation is a technique used to control behavior during pediatric dental rehabilitation, to reduce anxiety and to improve patient behavior. This facilitates painful procedures while ensuring safety and comfort without compromising the airways. The level of evidence regarding the efficacy and safety of sedation in pediatric dentistry is acceptable, with a total of 67% of moderate to high quality SRs. The most recommended, with the highest level of confidence, is the combination of N_2_O (30–50%) with midazolam, with an optimal dose of oral midazolam of 0.75 mg/kg. Based on the results of this umbrella review, future SRs should increase methodological consistency.

## Figures and Tables

**Table 1:** Studies’ characteristics.

Author/Year	Seartch Period	Focus	Electronic Databases	Sample Size (Patients)	Method of Analysis	Tool Used for Quality Assessment	Types/No of Studies Included	Intervention/ Dose	Adverse Effects Reported	Outcomes	AMSTAR2	Funding
Fu et al. (2023) [11]	Up to April, 2022	G	PubMed, Embase, Cochrane Libary,	114	SR & MA	Cochrane ROB	2 RCTs	IN DEX (1 µg/kg) vs. IN MDZ (0.2 mg/kg)	Yes	Parental separation, satisfactory anesthesia induction or facemask acceptance, Postoperative pain and agitation, Sedation Level, Onset of sedation, Recovery time, Hemodynamic status, Adverse events	High	No
Fatani et al. (2022) [12]	Unclear	D	PubMed, Cochrane Libary, WoS, Google Scholar	1019	SR	None	10	MDZ Vs Routes of administration (oral, sublingual, intravenous, intranasal)	No	Safety, efficacy an compare the different routes of administration.	Critically Low	NR
Kotian et al. (2022) [13]	Unclear	D	Pubmed, Cochrane Libary, Science Direct, Sigle, LILACS, Google Scholar	201	SR	Cochrane database	4 RCTs	IN MDZ (0.1–0.3 mg/kg) vs. Oral MDZ (0.2–0.7 mg/kg)	No	Sedation Level, Behaviour rating and overall success rate	Moderate	No
Lang et al. (2022) [14]	Up to 20 April 2022	G	Pubmed, Embase, Cochrane Library	62	SR & MA	Cochrane ROB	2 RCTs	IN MDZ (0.2–0.4 mg/kg) vs. IN Ketamine (3–5 mg/kg)	Yes	Sedative effect, Recovery time, Adverse effects, Onset of sedation, Hemodynamic status	High	Science and Technology Plan Project of Sichuan Province
Lin et al. (2022) [15]	Up to October 2021	G	Pubmed, Embase, WoS, Cochrane Library	221	SR & MA	Cochrane ROB	3 RCTs	IN DEX vs. IN DEX + Ketamine vs. Oral MDZ vs. nebulized ketamine	Yes	Satisfactory sedative, onset of sedation, recovery time, adverse events, satisfactory mask acceptance, satisfactory separation from parents, emergence agitation.	High	NR
Mellor et al. (2022) [16]	Unclear	G	Medline, Embase, CENTRAL, WoS	83	SR	Cochrane ROB2	2 RCTs	Melatonin (0.5–3 mg/kg) vs. MDZ (0.5–0.75 mg/kg) vs. Placebo	Yes	Sedation Success, Parents and Operators satisfaction, side effects, physiologic parameters	Moderate	No
Oza et al. (2022) [4]	Up to 31 January 2020	D	Medline, Pubmed, CENTRAL, Cochrane	229	SR & MA	Cochrane ROB	3 RCTs	DEX (1–2 mg/kg, IN) vs. MDZ (0.2–5 mg/kg, IN and oral)	Yes	Level of sedation (Intraoperative and postoperative analgesia), recovery time and level of anxiolysis	High	No
Taneja & Jain (2022) [17]	Began in May 2020	D	Pubmed, MEDLINE, Google Scholar, Cochrane	367	SR & MA	Cochrane ROB	7 RCTs	DEX (oral, IN) vs. MDZ (oral, IN)	No	Anesthesia success rate, onset and duration, heart rate, blood pressure, oxygen saturation	High	NR
Goswami et al. (2021) [18]	Up to 20 May 2020	D	Pubmed (Medline), Google Scholar, Cochrane Library, SCOPUS, LILACS	418	SR & MA	ROBIS	6	DEX (1–2 µg/kg; IV and IN) vs. MDZ (0.05–0.5 mg/kg; oral, IN; IV)	Yes	Quality of separation from parents, effect on behaviour management, time of onset, recovery time, success rate and level of sedation, effect on vital parameters, postoperative nausea and vomiting, shivering, emergence delirium, mask acceptance	Low	NR
Hayes et al. (2021) [19]	Up to March, 2019	G	Medline, Embase, CENTRAL, Cochrane Database os Systematic Reviews, WoS	160	SR & MA	RoB	3 RCTs	Propofol (1–1.5 mg/kg, IV) vs. Ketofol (0.5–1.5 mg/kg propofol + 0.5–0.25 mg/kg ketamine) vs. ketamine (1 mg/kg) + DEX (0.5 µg/kg) vs. ketamine (1 mg/kg)	Yes	Physiological parameters, recovery time, intraoperative and postoperative adverse events, success of procedure, operator satisfaction, sedation quality, treatment time.	High	Institutional and/or departmental
Preethy & Somasundara m (2021) [1]	Up to December, 2019	D	Cochrane, Pubmed, LILACS, Science Direct, Google Scholar, SIGLE	1309	SR	Cochrane database	13 RCTs	IN MDZ (0.1–0.5 mg/kg) vs. Other administrative routes of MDZ (oral (0.2–1 mg/kg), sublingual (0.2–0.3 mg/kg), IM (0.2 mg/kg))	No	Sedative effect, Effect on anxiety and behaviour, Success rate	Moderate	No
Rossit et al. (2021) [20]	Up to 2 April 2021	D	Pubmed, CENTRAL, Scopus, EBSCO, LILACS, Summon, DARE	1098	SR & MA	RoB 2	19 (8 RCTs and 11 crossover trials)	NOIS (< or = 50% N_2_O) vs. other drugs or sedation tecniques or placebo	NR	Clinical success rate of NOIS and create a dataset of efficacy criteria prevalence in published trials.	Low	NR
Cheng et al. (2020) [21]	Up to August, 2018	G	Pubmed, Embase, Cochrane Library, CINAHL, International Pharmaceuticals, four chinese electronic databases	1070	SR & MA	Cochrane ROB	12	MDZ (0.5 mg/kg) vs. MDZ (0.75 mg/kg) vs. MDZ (1 mg/kg) vs. Cloral hydrate (50 mg/kg) vs. MDZ + Meperidine (0.7 mg/kg)	Yes	Behaviour, Physiological parameters, overall sedation success rate, acceptance of medication and local anesthesia, level of anxiety and sedation, safety and effectiveness of sedation, adverse effects, duration of action, parents satisfaction	High	Key Project of Sichuan Provincial Health Commission, key project of Sichuan Provincial Provincial Department of Science and Technology, and the National Science and Technology Major Special Project.
Foo et al. (2020) [22]	Up to February, 2019	G	CENTRAL, Medline	75	SR & MA	Unclear	1 RCT	Ketofol (0.6 mg/kg, continuous infusion 40–60 µg/kg/min) vs. Ketamine (1 mg/kg, continuous infusion 50–60 µg/kg/min ) vs. Propofol (2 mg/kg, continuous infusion 70–90 µg/kg/min)	Yes	Recovery time, Adverse effects, Hemodynamic parameters	Low	No
Lang et al. (2020) [23]	Up to 2019 October	G	Pubmed, Embase, Cochrane Library	251	SR & MA	Cochrane ROB	4 RCTs	DEX (oral (4 µg/kg), IN ( 0.2–1.5 µg/kg)) vs. MDZ (oral (0.5 mg/kg), IN (0.2–0.5 mg/kg))	Yes	Separation from parents, induction or mask acceptance, postoperative pain, Emergence agitation, Hemodynamics status (SBP, DBP, HR), Onset of sedation, recover time, Adverse events	High	NR
Poonai et al. (2020) [24]	Up to July, 2019	G	Medline, Embase, Scopus, WoS, Google Scholar, Cochrane Central Register, CINAHL	128	SR	Cochrane ROB	2 RCTs	IN DEX (1–2.5 µg/kg) vs. Oral DEX (4–5 µg/kg) vs. IN MDZ (0.2 mg/kg) vs. IN Ketamine (5 mg/kg)	Yes	Onset of sedation, Adequate depth of sedation, Recovery time, Treatment outcome, acceptance of drug administration, ease of treatment completion, hemodynamic status, success rate, intra/post-operative analgesia, adverse effects	Moderate	No
Tervonen et al. (2020) [25]	Up to December 2019	G	Pubmed, Scopus, Science Direct, Cochrane Library, ClinicalTrials.gov	63	SR & MA	Cochrane ROB	1 RCT	DEX (1 µg/kg) vs. DEX (1.5 µg/kg) vs. IN MDZ (0.2 mg/kg)	Yes	Success rate, onset time and duration of sedation, time to discharge and occurrence of adverse events	Low	Finnish foundation for Pediatric Research and Alma and KA Snellman Foundation
Chen et al. (2019) [26]	Up to September, 2018	G	Pubmed, Embase, Cochrane library, China National Knowledge infrastructure, WanFang Database, Chinese Biomedical Literature Database, VIP Database for Chinese Technical Periodicals	274	SR & MA	Cochrane ROB	3 RCTs	Oral Chloral hydrate (75 mg/kg) vs. Placebo OR Oral Chloral hydrate (40 mg/kg) vs. Oral Diazepam (5 mg) OR Oral chloral hydrate (0.8–1.0 mL/ug) vs. Intramuscular Phenobarbital (5 mg/kg)	No	Success rate of sedation	Moderate	NR
Hu et al. (2019) [27]	Up to July, 2018	G	Cochrane Library, Pubmed, Medline, Embase, China National Knowledge Infrastructure	90	SR & MA	Cochrane ROB	2 RCTs	Ketofol (0.6 mg/kg or 0.5 mg/kg K + 0.5 mg/kg P) vs. Ketamine (1 mg/kg)	Yes	Adverse events, time endpoints (procedure time, recovery time, sedation time), satisfaction of patients/providers	Moderate	No
Kim et al. (2019) [28]	Up to January 31, 2018	G	Medline, Embase, CENTRAL	210	SR & MA	Unclear	4 RCTs	Propofol vs. Other drugs (dexmedetomi dine, ketamine, midazolam)	Yes	Physiological parameters, Onset of sedation, Recovery time, Adverse Effects, duration of the action, Behaviour, Procedure time, Discharge time	Critically Low	NR
Manso et al. (2019) [29]	Up to March, 2016	G	Pubmed, Science Direct	180	SR	Unclear	5 RCTs	Oral MDZ (0.3–0.5 mg/kg) vs. Oral Ketamine (5 mg/kg) vs. Hydroxyzine (3.7 mg)+ MDZ (0.3 mg/Kg) vs. Nitrous oxide (30% in oxygen)	Yes	Physiological parameters, Sedation and behaviour scores, effectiveness, anxiety levels and postoperative satisfaction, overall safety and success	Critically Low	Advicenne
Qiu & Luo (2019) [30]	Up to June 2018	D	Pubmed, Embase and CENTRAL	163	SR & MA	Cochrane ROB	4 RCTs	DEX (1–5 µg/kg) vs. Ketamine (2–8 mg/kg)	No	Sedation level, intraoperative and postoperative analgesia, heart rate, SBP, DBP, SpO2 and recovery time.	High	NR
Ashley et al. (2018) [2]	Up to February 22, 2018	D	Cochrane Oral Health´s Trials Register, CENTRAL, Medline, Embase	3704	SR & MA	Cochrane ROB	50 RCTs	Sedative vs. Placebo OR Sedative vs. different dosage OR Sedative vs. Other sedatives	Yes	Behaviour, Completion of treatment, Postoperative anxiety, adverse events	High	Supported by the NIHR, via Cochrane Infrastructure funding to Cochrane Oral Health.
Oh & Kingsley (2018) [31]	Up to December 31, 2017	G	Pubmed	1425	SR	Unclear	9	Retal administration vs. other routes	Yes	Sedation effectiveness	Critically Low	NR
Poonai et al. (2018) [32]	Unclear	D	Pubmed and Medline	1331	SR	None	25 trials	Ketamine (alone or in combination) vs. other drugs vs. administration route	No	Sedation score, % good sedation and time to Effect	Critically Low	NR
Feng et al. (2017) [33]	Up to August, 2018	G	Medline, Embase, WoS, Scopus, CINAHL, Google Scholar, Cochrane Library	95	SR	Cochrane ROB	2 RCTs	IN Ketamine (6 mg/kg) vs. IN MDZ (0.3 mg/kg) vs. IN MDZ (0.2–0.3 mg/kg) +ketamine (4–5 mg/kg) vs. IN sufentanil (20µg) + MDZ (0.3 mg/kg)	Yes	Safety, efficacy and overall success rate of sedation, adverse effects	Moderate	NR
Jun et al. (2017) [34]	Up to December 2016	G	Pubmed, Ovid, WoS, Public Health Management Corporation	113	SR & MA	Cochrane ROB	2 RCTs	Oral DEX (4 µg/kg) vs. Oral MDZ (0.5 mg/kg) OR IN DEX (1 µg/kg) vs. IN MDZ (0.2 mg/kg)	Yes	Separation from parents, mask acceptance, effectiveness in reducing the incidence and severity of Emergence delirium, postoperatory pain, agitation and shivering, onset of sedation, recovery time, Adverse events	High	Guangxi Natural Science Foundation of China
Kim et al. (2017) [35]	Up to November 3, 2016	G	Medine, Embase, CENTRAL, WoS, Google Scholar, KoreaMed	72	SR & MA	Cochrane ROB	1 RCT	IN MDZ (0.2 mg/Kg) vs. IN DEX (1 µg/kg)	Yes	Sedation status ate separation from parents, onset of sedation, mask acceptance, recovery times, postoperative agitation, postoperative pain.	High	No
Kuang et al. (2017) [36]	Up to December 31, 2014	G	Medline, Embase, Cochrane Library, KoreaMed	72	SR & MA	Cochrane ROB	1 RCT	IN DEX (1.0 µg/kg) vs. IN MDZ (0.2 mg/kg)	Yes	Sedation Score	High	No
Poonai et al. (2017) [37]	Up to August 22, 2016	G	Pubmed	32	SR & MA	Unclear	1 RCT	Oral MDZ (7.5 mg) vs. Placebo	No	Mental attitude, hynotic and motor activity, quality of sedation and dental treatment.	Critically Low	No
Sivaramakris hnan & Sridharan ( 2017) [38]	Up to August, 2016	G	Medline, Embase, Google Scholar, CINAHL, Cochrane library, WoS, Scopus, clinical trial registries, conference proceedings	216	SR	Cochrane ROB	5 RCTs	IN Ketamine (3 a 10 mg/kg) vs. Other drugs and routes of administration (Sufentanil, MDZ, Ketamine + MDZ, DEX)	Yes	Efectiveness of sedation, onset and duration of sedation, ease of administration, analgesia, additional sedative medication, adverse effects.	Moderate	No
ter Bruggen et al. (2016) [39]	Up to February 20, 2016	D	Medline, CENTRAL, DARE	2378	SR & MA	Cochrane ROB	4 RCTs	NOIS (30–50% N_2_O) + MDZ (i.v 0.05–2 mg) vs. NOIS or MDZ (0.05 a 0.6 mg) alone	Yes	Overall cooperation during Treatment, Total dose, recovery time and adverse effects	High	NR
Pasin et al. (2015) [40]	Up to March, 2014	G	Embase, Medline, WoS Scopus, Cochrane, Pubmed, Google Scholar	60	SR	Unclear	1 RCT	IV DEX (2µg/kg followed by 4µg/kg continuous infusion) vs. IV MDZ (0.05 mg/Kg)- propofol (1 mg/Kg followed by 5 mg/kg/h continuous infusion)	Yes	Duration of procedure, Recovery time, Heart rate, Peripheral capillary oxygen saturation, mean arterial pressure	Critically Low	NR
Peng et al. (2014) [41]	Up to August 15, 2014	G	Pubmed, BioMedCentral, Embase, CENTRAL	113	SR & MA	Cochrane ROB	2 RCTs	Oral DEX (4 µg/kg) vs. Oral MDZ (0.5 mg/kg) OR IN DEX (1 µg/kg) vs. IN MDZ (0.2 mg/kg)	Yes	Separation from parents, mask acceptance, effectiveness in reducing the incidence and severity of Emergence delirium, postoperatory pain, agitation and shivering, onset of sedation, recovery time, Adverse events	High	No
Sun et al. (2014) [42]	Up to April, 2014	G	PubMED, Cochrane Library, Embase	113	SR & MA	Cochrane ROB	2 RCTs	Oral DEX ( 4 µg/kg) vs. Oral MDZ (0.5 mg/kg) OR IN DEX (1 µg/kg) vs. IN MDZ (0.2 mg/kg)	Yes	Separation from parents, mask acceptance, effectiveness in reducing the incidence and severity of Emergence delirium, postoperatory pain, agitation and shivering, onset of sedation, recovery time, Adverse events	Moderate	NR
Pedersen et al. (2013) [43]	Up to December 16, 2013	G	PubMED, Cochrane Library Ovid, Google Scholar	113	SR & MA	Cochrane ROB	2 RCTs	Oral DEX ( 4 µg/kg) vs. Oral MDZ (0.5 mg/kg) OR IN DEX (1 µg/kg) vs. IN MDZ (0.2 mg/kg)	Yes	Separation from parents, mask acceptance, effectiveness in reducing the incidence and severity of Emergence delirium, postoperatory pain, agitation and shivering, onset of sedation, recovery time, Adverse events	High	National Natural Science Foundation of China
Patel et al. (2018) [44]	Unclear	G	PubMED, Cochrane Library	1205	SR	Unclear	1 Observational multicentre study	N_2_O/O_2_ vs. other pharmacologic al treatments	Yes	Safety and efficacy of Nitrous oxide	Critically Low	NR

Characteristics of the included studies. NR—not reported, DEX—dexmedetomidine, MDZ—midazolam, IN—intranasal route, IV—intravenous route, IM—intramuscular route, RCT—randomized controlled trials, N_2_O/NOIS—nitrous oxide, SBP—systolic blood pressure; DBP—Diastolic blood pressure, HR—Heart rate, SpO_2_—oxygen saturation, SR—systematic review, MA—meta-analysis, AMSTAR 2—“Measurement Tool to Assess Systematic Reviews, *”* PRISMA—“Preferred Reporting Items for Systematic Reviews and Meta-analysis,” CENTRAL—“Cochrane Central Register of Controlled Trials,” CINAHL—“Cumulative index to nursing and allied health literature,” LILACS—Literatura Latino-Americana e do Caribe em Ciências da Saúde, WoS—“Web of Science,” DARE—“Database of Abstracts of Reviews of Effects”.

**Table 2:** Summary of the AMSTAR2 results per item and article.

Author/Year	1	2	3	4	5	6	7	8	9	10	11	12	13	14	15	16	Overall
Fu et al. (2023) [11]	Y	PY	Y	Y	Y	Y	Y	PY	PY/0	N	Y/0	Y	Y	Y	Y	Y	High
Fatani et al. (2022) [12]	Y	N	N	N	N	Y	PY	N	N/N	N	0/0	0	N	N	0	N	Critically Low
Kotian et al. (2022) [13]	Y	PY	Y	PY	N	N	Y	PY	PY/0	N	0/0	0	Y	N	0	Y	Moderate
Lang et al. (2022) [14]	Y	PY	Y	PY	Y	Y	Y	PY	PY/0	N	Y/0	Y	Y	Y	Y	Y	High
Lin et al. (2022) [15]	Y	PY	Y	PY	Y	Y	Y	PY	PY/0	N	Y/0	Y	Y	Y	Y	Y	High
Mellor et al. (2022) [16]	Y	Y	Y	PY	Y	Y	Y	PY	PY/0	N	0/0	0	Y	Y	0	Y	Moderate
Oza et al. (2022) [4]	Y	PY	Y	PY	Y	Y	Y	PY	PY/0	N	Y/0	Y	Y	Y	Y	Y	High
Taneja and Jain (2022) [17]	Y	PY	Y	PY	Y	Y	Y	PY	PY/0	N	Y/0	Y	Y	Y	Y	Y	High
Goswami et al. (2021) [18]	Y	PY	N	PY	Y	Y	Y	PY	N/0	N	0/0	0	Y	Y	0	Y	Low
Hayes et al. (2021) [19]	Y	PY	Y	Y	Y	Y	Y	PY	PY/0	N	Y/0	Y	Y	Y	Y	Y	High
Preethy and Somasundaram (2021) [1]	Y	PY	Y	PY	N	Y	Y	PY	PY/0	N	0/0	0	Y	N	0	Y	Moderate
Rossit et al. (2021) [20]	Y	PY	Y	PY	Y	Y	Y	PY	N/0	N	Y/0	Y	Y	Y	Y	Y	Low
Cheng et al. (2020) [21]	Y	PY	Y	PY	Y	Y	PY	PY	PY/PY	N	Y/Y	Y	Y	Y	Y	Y	High
Foo et al. (2020) [22]	Y	PY	Y	PY	Y	Y	PY	PY	PY/0	N	N/0	Y	Y	Y	Y	Y	Low
Lang et al. (2020) [23]	Y	Y	Y	PY	Y	Y	Y	PY	PY/0	N	Y/0	Y	Y	Y	Y	Y	High
Poonai et al. (2020) [24]	Y	PY	Y	PY	Y	Y	Y	PY	PY/0	N	0/0	0	Y	Y	0	Y	Moderate
Tervonen et al. (2020) [25]	Y	PY	Y	PY	Y	Y	Y	PY	N/0	N	Y/0	Y	Y	N	Y	Y	Low
Chen et al. (2019) [26]	Y	PY	Y	PY	Y	Y	Y	PY	PY/0	N	Y/0	Y	Y	Y	Y	Y	Moderate
Hu et al. (2019) [27]	Y	PY	Y	PY	Y	Y	PY	PY	PY/0	N	Y/0	Y	Y	Y	Y	N	Moderate
Kim et al. (2019) [28]	Y	N	Y	N	N	N	N	PY	PY/0	N	Y/0	Y	Y	Y	Y	Y	Critically Low
Manso et al. (2019) [29]	Y	N	Y	PY	N	N	Y	PY	N/0	N	0/0	0	N	Y	0	Y	Critically Low
Qiu and Luo (2019) [30]	Y	PY	Y	PY	Y	Y	Y	PY	PY/0	N	Y/0	Y	Y	Y	Y	Y	High
Ashley et al. (2018) [2]	Y	Y	Y	Y	Y	Y	Y	Y	Y/0	N	Y/0	Y	Y	Y	Y	Y	High
Oh and Kingsley (2018) [31]	Y	N	Y	N	Y	Y	PY	PY	N/N	N	0/0	0	N	N	0	N	Critically Low
Poonai et al. (2018) [32]	Y	N	Y	PY	Y	Y	PY	PY	N/0	N	0/0	0	N	N	0	Y	Critically Low
Feng et al. (2017) [33]	Y	PY	Y	PY	Y	Y	Y	PY	PY/0	N	0/0	0	Y	Y	0	Y	Moderate
Jun et al. (2017) [34]	Y	PY	Y	Y	Y	Y	PY	PY	PY/0	N	Y/0	Y	Y	Y	Y	Y	High
Kim et al. (2017) [35]	Y	PY	Y	Y	Y	Y	Y	PY	PY/0	N	Y/0	Y	Y	Y	Y	Y	High
Kuang et al. (2017) [36]	Y	PY	Y	PY	Y	Y	Y	PY	PY/0	N	Y/0	Y	Y	Y	Y	Y	High
Poonai et al. (2017) [37]	Y	PY	Y	N	N	N	N	PY	N/0	N	Y/0	Y	Y	Y	Y	Y	Critically Low
Sivaramakris hnan and Sridharan (2017) [38]	Y	PY	Y	PY	Y	Y	Y	PY	PY/0	N	0/0	0	Y	Y	0	Y	Moderate
ter Bruggen et al. (2016) [39]	Y	PY	Y	PY	Y	Y	Y	PY	PY/0	N	Y/0	Y	Y	Y	Y	Y	High
Pasin et al. (2015) [40]	Y	N	Y	N	Y	Y	Y	PY	N/0	N	0/0	0	N	N	0	Y	Critically Low
Peng et al. (2014) [41]	Y	PY	Y	PY	Y	Y	Y	PY	PY/0	N	Y/0	Y	Y	Y	Y	Y	High
Sun et al. (2014) [42]	Y	PY	Y	Y	Y	Y	PY	PY	PY/0	N	Y/0	Y	Y	Y	Y	N	Moderate
Pedersen et al. (2013) [43]	Y	PY	Y	PY	Y	Y	Y	PY	Y/0	N	Y/0	Y	Y	Y	Y	Y	High
Patel et al. (2018) [44]	Y	N	Y	PY	N	N	PY	PY	N/0	N	0/0	0	N	N	0	Y	Critically Low

“Y” = yes; “N” = no; “PY” = partial yes; “0” = not applicable. 1. Are there research questions and do they meet the inclusion criteria? 2. Are review methods established a priori? 3. Is there an explanation of their selection literature search strategy? 4. Did the review authors use a comprehensive literature search strategy? 5. Was the study selection performed in duplicate? 6. Was data selection performed in duplicate? 7. Was the list of excluded studies and exclusions justified? 8. Is the description of the included studies in adequate detail? 9. Is there a satisfactory technique for assessing the risk of bias (RoB)? 10. Is a report on the sources of funding for the studies included in the review? 11. If meta-analysis was performed, did the review authors use appropriate methods for statistical combination of results? 12. If meta-analysis was performed, did the review authors assess the potential impact of RoB? 13. Was RoB accounted for when interpreting/discussing the results of the review? 14. Did the review authors provide a satisfactory explanation for, and discussion of, any heterogeneity observed in the results of the review? 15. If quantitative synthesis was performed, was publication bias performed? 16. Did the review authors report any potential sources of conflict of interest, including funding sources?

**Table 3:** Summary of route and dosage in sedation with references.

Active Principle	Route	Dosage	Level of Evidence Based on RoB	References
Midazolam	Oral	0.3 mg/Kg	Critically low	[29]
	Oral	0.5 mg/Kg	Critically low	[29]
	Oral	0.75 mg/Kg	High	[21]
Dexmedetomidine	Intranasal	2.5 μg/Kg		[24,45]
	Intranasal	1.5 μg/kg		[24,46]

**Table 4:** Summary of adverse effects in sedation, with references.

Adverse Effect	References
**Most Commonly Reported**	
Vomiting	[2,4,15,16,19,22,23,27,28,29,33,34,35,38,41,42,43,44]
Nausea	[2,15,16,19,21,22,23,27,28,34,35,38,41,42,43,44]
Nasal irritation (when in intranasal administration)	[2,4,11,18,23,34,35,36,41,42,43]
Teary eyes	[4,11,23,34,35,41,42,43]
Shivering	[18,23,34,35,41,42,43]
Agitation	[2,18,22,27,38,44,49]
Cough	[2,16,19,22,27,28]
Diplopia	[2,19,21,22,27]
Drowsiness	[2,21,29,49]
Desaturation	[15,19,38,49]
Hypoxia	[2,19,28]
Amnesia	[2,16,49]
Hallucinations, hypersalivation and hiccups	[2,22,27]
Enuresis	[2,16]
Headache	[29,44]
Pain at injection site and spontaneous movement	[22,27]
**Least Commonly Reported**	
Dysphoria and respiratory events	[21]
Pyrexia	[28]
Postoperative pain, nightmares and paradoxical reaction	[29]
Apnea, stridor and laryngospasm	[19]
Aggressiveness and tiredness	[49]
Tremors and dizziness	[16]
Airway problems, bronchospasm, otalgia, irritability, sleepiness and sneezing	[2]
Pallor, vertigo and sweating	[44]

## Data Availability

Data are freely available throughout the Supplementary Files related to this work.

## Supplementary Materials

The following supporting information can be downloaded at: https://www.mdpi.com/article/10.3390/jcm13123544/s1, Table S1: PRISMA checklist; Table S2: List of excluded studies with reasons.
