# Supplementary material for: Evaluating the Quality of Systematic Reviews on Pediatric Sedation in Dentistry: An Umbrella Review
Source: J Clin Med. 2024 Jun 17;13(12):3544. doi: 10.3390/jcm13123544 (PMC11205123; doi:10.3390/jcm13123544)
Supplement: Supplementary file 1 [file jcm-13-03544-s001.zip › jcm-3047526-supplementary.pdf]

Supplementary Material S1. PRISMA Checklist

Section and Topic	Item #	Checklist item	Location where item is reported
**TITLE**			
Title	1	Identify the report as a systematic review.	1
**ABSTRACT**			
Abstract	2	See the PRISMA 2020 for Abstracts checklist.	1
**INTRODUCTION**			
Rationale	3	Describe the rationale for the review in the context of existing knowledge.	1
Objectives	4	Provide an explicit statement of the objective(s) or question(s) the review addresses.	1
**METHODS**			
Eligibility criteria	5	Specify the inclusion and exclusion criteria for the review and how studies were grouped for the syntheses.	2
Information sources	6	Specify all databases, registers, websites, organisations, reference lists and other sources searched or consulted to identify studies. Specify the date when each source was last searched or consulted.	2
Search strategy	7	Present the full search strategies for all databases, registers and websites, including any filters and limits used.	2
Selection process	8	Specify the methods used to decide whether a study met the inclusion criteria of the review, including how many reviewers screened each record and each report retrieved, whether they worked independently, and if applicable, details of automation tools used in the process.	2
Data collection process	9	Specify the methods used to collect data from reports, including how many reviewers collected data from each report, whether they worked independently, any processes for obtaining or confirming data from study investigators, and if applicable, details of automation tools used in the process.	3
Data items	10a	List and define all outcomes for which data were sought. Specify whether all results that were compatible with each outcome domain in each study were sought (e.g. for all measures, time points, analyses), and if not, the methods used to decide which results to collect.	3
	10b	List and define all other variables for which data were sought (e.g. participant and intervention characteristics, funding sources). Describe any assumptions made about any missing or unclear information.	3
Study risk of bias assessment	11	Specify the methods used to assess risk of bias in the included studies, including details of the tool(s) used, how many reviewers assessed each study and whether they worked independently, and if applicable, details of automation tools used in the process.	3
Effect measures	12	Specify for each outcome the effect measure(s) (e.g. risk ratio, mean difference) used in the synthesis or presentation of results.	3
Synthesis methods	13a	Describe the processes used to decide which studies were eligible for each synthesis (e.g. tabulating the study intervention characteristics and comparing against the planned groups for each synthesis (item #5)).	NA
	13b	Describe any methods required to prepare the data for presentation or synthesis, such as handling of missing summary statistics, or data conversions.	NA

Section and Topic	Item #	Checklist item	Location where item is reported
	13c	Describe any methods used to tabulate or visually display results of individual studies and syntheses.	NA
	13d	Describe any methods used to synthesize results and provide a rationale for the choice(s). If meta-analysis was performed, describe the model(s), method(s) to identify the presence and extent of statistical heterogeneity, and software package(s) used.	NA
	13e	Describe any methods used to explore possible causes of heterogeneity among study results (e.g. subgroup analysis, meta-regression).	NA
	13f	Describe any sensitivity analyses conducted to assess robustness of the synthesized results.	NA
Reporting bias assessment	14	Describe any methods used to assess risk of bias due to missing results in a synthesis (arising from reporting biases).	NA
Certainty assessment	15	Describe any methods used to assess certainty (or confidence) in the body of evidence for an outcome.	NA
**RESULTS**			
Study selection	16a	Describe the results of the search and selection process, from the number of records identified in the search to the number of studies included in the review, ideally using a flow diagram.	3
	16b	Cite studies that might appear to meet the inclusion criteria, but which were excluded, and explain why they were excluded.	3
Study characteristics	17	Cite each included study and present its characteristics.	4
Risk of bias in studies	18	Present assessments of risk of bias for each included study.	4
Results of individual studies	19	For all outcomes, present, for each study: (a) summary statistics for each group (where appropriate) and (b) an effect estimate and its precision (e.g. confidence/credible interval), ideally using structured tables or plots.	4
Results of syntheses	20a	For each synthesis, briefly summarise the characteristics and risk of bias among contributing studies.	15
	20b	Present results of all statistical syntheses conducted. If meta-analysis was done, present for each the summary estimate and its precision (e.g. confidence/credible interval) and measures of statistical heterogeneity. If comparing groups, describe the direction of the effect.	NA
	20c	Present results of all investigations of possible causes of heterogeneity among study results.	NA
	20d	Present results of all sensitivity analyses conducted to assess the robustness of the synthesized results.	NA
Reporting biases	21	Present assessments of risk of bias due to missing results (arising from reporting biases) for each synthesis assessed.	NA
Certainty of evidence	22	Present assessments of certainty (or confidence) in the body of evidence for each outcome assessed.	NA
**DISCUSSION**			
Discussion	23a	Provide a general interpretation of the results in the context of other evidence.	19

Section and Topic	Item #	Checklist item	Location where item is reported
	23b	Discuss any limitations of the evidence included in the review.	19
	23c	Discuss any limitations of the review processes used.	20
	23d	Discuss implications of the results for practice, policy, and future research.	20
**OTHER INFORMATION**			
Registration and protocol	24a	Provide registration information for the review, including register name and registration number, or state that the review was not registered.	2
	24b	Indicate where the review protocol can be accessed, or state that a protocol was not prepared.	2
	24c	Describe and explain any amendments to information provided at registration or in the protocol.	22
Support	25	Describe sources of financial or non-financial support for the review, and the role of the funders or sponsors in the review.	22
Competing interests	26	Declare any competing interests of review authors.	22
Availability of data, code and other materials	27	Report which of the following are publicly available and where they can be found: template data collection forms; data extracted from included studies; data used for all analyses; analytic code; any other materials used in the review.	22

From: Page MJ, McKenzie JE, Bossuyt PM, Boutron I, Hoffmann TC, Mulrow CD, et al. The PRISMA 2020 statement: an updated guideline for reporting systematic reviews. BMJ 2021;372:n71. doi: 10.1136/bmj.n71

For more information, visit: http://www.prisma-statement.org/

Supplementary Material S2. Excluded studies with reasons

Nº	References	Reason
1	Lu, C., Zhang, Y. Y., Xiang, B., Peng, S., Gu, M., & Wong, H. M. (2023). Management of fear and anxiety in dental treatments: A systematic review and meta-analysis of randomized controlled trials. *Odontology*, *111*(1), 20–32. https://doi.org/10.1007/s10266-022-00711-x	Unrelated
2	Delvi, M. B. (2022). Is intranasal dexmedetomidine superior to oral chloral hydrate for procedural sedation in children: A systematic review. *Saudi Journal of Anaesthesia*, *16*(1), 82–85. Scopus. https://doi.org/10.4103/sja.sja_489_21	Unrelated
3	Flores-Pérez, C., Moreno-Rocha, L. A., Chávez-Pacheco, J. L., Noguez-Méndez, N. A., Flores-Pérez, J., Alcántara-Morales, M. F., Cortés-Vásquez, L., & Sarmiento-Argüello, L. (2022). Sedation level with midazolam: A pediatric surgery approach. *Saudi Pharmaceutical Journal*, *30*(7), 906–917. https://doi.org/10.1016/j.jsps.2022.05.002	Literature Review
4	Gao, P., Li, S., Li, Y., Zhao, L., Luo, Q., & Ji, Y. (2022). The comparison of ketamine-dexmedetomidine (ketadex) and ketamine-propofol (ketofol) for procedural sedation in pediatric patients: A meta-analysis of randomized controlled trials. *Heliyon*, *8*(10), e11166. https://doi.org/10.1016/j.heliyon.2022.e11166	Unrelated
5	Suhael Ahmed, Norah Khalid Alali, Ali Salem Alsalem, Naif Saleh Alosily, Omar Abdulkarim Altorbaq, Mashael Alsadoon, Ahmed Al Ghamdi, Evaluation of Safety of Using Sedative Anesthesia in Pediatric Dentistry: A Systematic Review, J Res Med Dent Sci, 2022, 10 (9):166-172	Unrelated
6	Wang, J., Li, Y., Xiao, S., Shi, B., Xia, Z., Huang, C., Xu, H., Li, N., & Tang, W. (2022). Efficacy and Safety of Intranasal Dexmedetomidine versus Oral Chloral Hydrate as Sedatives for Pediatric Patients: A Systematic Review and Meta-Analysis. *Journal of Investigative Medicine*, *70*(5), 1219–1224. https://doi.org/10.1136/jim-2021-002038	Not available
7	Procaccini, D., Lobner, K., Azamfirei, R., & Kudchadkar, S. R. (2021). Melatonin for anaesthetic indications in paediatric patients: A systematic review. *Anaesthesia*, *76*(6), 837–849. https://doi.org/10.1111/anae.15249	Unrelated
8	De Stefano, R., Bruno, A., Muscatello, M. R., Cedro, C., Cervino, G., & Fiorillo, L. (2020). Fear and anxiety managing methods during dental treatments: A systematic review of recent data. Minerva Stomatologica, 68(6). https://doi.org/10.23736/S0026-4970.19.04288-2	Not available

9	Lewis, J., & Bailey, C. (2020). Intranasal dexmedetomidine for sedation in children; a review. Journal of Perioperative Practice, 30(6), 170–175. https://doi.org/10.1177/1750458919854885	Literature review
10	Long, Y., Zhang, C., Tan, C., Zhang, J., & Wang, Z. (2020). Efficacy of intranasal dexmedetomidine versus oral midazolam for premedication in pediatric patients: A meta-analysis. *Chinese Journal of Anesthesiology*, *40*(2), 195–198. Scopus. https://doi.org/10.3760/cma.j.issn.0254-1416.2020.02.017	Not available
11	Pérez-García, S., Lozano-Carrascal, N., Ruiz-Roca, J. A., López-Jornet, P., & Gargallo-Albiol, J. (2020). Evaluation of endovenous sedation using BIS monitoring in dentistry. A systematic review. *Medicina Oral, Patología Oral y Cirugía Bucal*, *25*(4), e439–e448. https://doi.org/10.4317/medoral.22884	Unrelated
12	Canton, K. R., Hendrikx, S., Joubert, G., Shah, A., Rieder, M., Lynch, T., & Poonai, N. (2018). Intranasal ketamine for procedural sedation and analgesia in children: A systematic review. Pediatrics, 141(1).	Current Update
13	Cianetti, S., Lombardo, G., Lupatelli, E., Pagano, S., Abraha, I., Montedori, A., Caruso, S., Gatto, R., De Giorgio, S., & Salvato, R. (2017). Dental fear/anxiety among children and adolescents. A systematic review. *European Journal of Paediatric Dentistry*, *18*(2), 121–130. https://doi.org/10.23804/ejpd.2017.18.02.07	Unrelated
14	Kim, S., Jang, M., Choi, Y., Hong, H., Lee, J., Kim, H., & Hahn, S. (2017). Evaluation of Safety and Efficacy of Propofol for Procedural Sedation in Pediatric Population: A Meta-Analysis. *Value in Health*, *20*(9), A544. https://doi.org/10.1016/j.jval.2017.08.827	Not available
15	Canton, K., Hendrikx, S., Joubert, G., Shah, A., Rieder, M., & Poonai, N. (2016). Intranasal ketamine for procedural sedation and analgesia in children: A systematic review. Annals of Emergency Medicine, 68(4), S73-S74. (Supplement 1).	Current Update
16	Ashley, P. F., Williams, C. E., Moles, D. R., & Parry, J. (2015). Sedation versus general anaesthesia for provision of dental treatment to patients younger than 18 years. Cochrane Database of Systematic Reviews, 2015(9). https://doi.org/10.1002/14651858.CD006334.pub4	Unrelated
17	Ashley, P. F., Williams, C. E., Moles, D. R., & Parry, J. (2012). Sedation versus general anaesthesia for provision of dental treatment in under 18 year olds. Em The Cochrane Collaboration (Ed.),	Unrelated

	Cochrane Database of Systematic Reviews (p. CD006334.pub3). John Wiley & Sons, Ltd. https://doi.org/10.1002/14651858.CD006334.pub3	
18	Lourenço-Matharu, L., Ashley, P. F., & Furness, S. (2012). Sedation of children undergoing dental treatment. *Cochrane Database of Systematic Reviews*. https://doi.org/10.1002/14651858.CD003877.pub4	Current Update
19	Ashley, P. F., Williams, C. E., Moles, D. R., & Parry, J. (2009). Sedation versus general anaesthesia for provision of dental treatment in under 18 year olds. Em The Cochrane Collaboration (Ed.), *Cochrane Database of Systematic Reviews* (p. CD006334.pub2). John Wiley & Sons, Ltd. https://doi.org/10.1002/14651858.CD006334.pub2	Unrelated
20	Matharu, L. L., & Ashley, P. F. (2007). What is the evidence for paediatric dental sedation? *Journal of Dentistry*, *35*(1), 2–20. https://doi.org/10.1016/j.jdent.2006.08.002	Literature review
21	Matharu, L., & Ashley, P. F. (2006). Sedation of anxious children undergoing dental treatment. Em The Cochrane Collaboration (Ed.), *Cochrane Database of Systematic Reviews* (p. CD003877.pub3). John Wiley & Sons, Ltd. https://doi.org/10.1002/14651858.CD003877.pub3	Current update
22	Matharu, L., & Ashley, P. (2005). Sedation of anxious children undergoing dental treatment. Em The Cochrane Collaboration (Ed.), *The Cochrane Database of Systematic Reviews* (p. CD003877.pub2). John Wiley & Sons, Ltd. https://doi.org/10.1002/14651858.CD003877.pub2	Current update
